# Supplementary material for: Patterns of Intron Gain and Loss in Fungi
Source: PLoS Biol. 2004 Nov 30;2(12):e422. doi: 10.1371/journal.pbio.0020422 (PMC532390; doi:10.1371/journal.pbio.0020422)
Supplement: Table S1 — Also available at http://genes.mit.edu/NielsenEtAl/. (4.3 MB ZIP). [file pbio.0020422.st001.zip › NielsenEtAl/html/642.html]

AN4101.1.NCU01852.1.MG03399.1.FG06579.1


```
 CLUSTAL W (1.82) Multiple Sequence Alignments - Introns Inserted


Sequence 1: NCU01852.1	396 aa
Sequence 2: MG03399.1	389 aa
Sequence 3: FG06579.1	373 aa
Sequence 4: AN4101.1	313 aa
Alignment Length: 396 aa
Number Identitical Residues: 168 aa
Alignment Score (without introns) 8841


MG03399.1 	MGINKESDNAAYMATTS------TAPREDIEPQLPNDRAATPDDNATGQRISAFKSLGWL
NCU01852.1	MTTKTVIPDASSMAEIGSSSESPTIPAKDAQPQDVEKQPSRETVNDRQKKVSAFKSLGLL
FG06579.1 	------------MSNTD------SPQTKESHDLENNTQPTEE-----KQGSSAFKALGWL
AN4101.1  	------------MNEEK----------HPRDPEAACATGCSPEADADDGPVSAFKQLGLL
          	            *              .  .             .      **** ** *

MG03399.1 	DRYLAVWCFLAMVLGVLLGNFVPETGPALQRGKFVGVSVAIA~IGLLVMMYPILCK~VRY
NCU01852.1	DRLLAVWIFLAMTVGVLLGNFVDNVGPALQKGTFVGVSIPIA~IGLLVMMYPILCK~VRY
FG06579.1 	DRLLAVWILLAMIIGILLGNFVPSTGAALEKGKFVGVSVPIA1VGLLVMMYPILCK~VRY
AN4101.1  	DRFLAVWIFLAMAVGIILGNFVPNTSETLQRGKFVDVSVPIA1VGLLVMMYPILCK0---
          	** **** :*** :*::***** ... :*::*.**.**:.** :************    

MG03399.1 	ESLHKLFAHRGVWKQIGFSVVVNWIIAPLLM~LALAWAFLPDKSELRSGLIMVGLGRCIA
NCU01852.1	ESLHELFSHRTIWKQIGFSILVNWIVAPFLM0LALSWAFLPDKEELRVGLILVGLGRCIA
FG06579.1 	ESLHEIFSQRDVWKQILFSIFINWIIAPFLM0LGLAWAFLPDKPELRVGLILVGLARCIA
AN4101.1  	-------------------------------~LGLAWAFLPDERGLREGLILVGIARCIA
          	                                *.*:******:  ** ***:**:.****

MG03399.1 	M0VLIWTNLAGGDEEYCAILVAVNSVLQMVLFAPLAVLFIGVISGENQGLEAKYTVVATS
NCU01852.1	M~VLIWTSLAGGDNEYCAILVAMNSILQMVLFAPLAVFFIRVISGESGVRDVSYEVVATS
FG06579.1 	M~VLIWNGLAGGDSEYCAILVAINSILQMALYAPMSVFFISIISGEEGSLEVSYSTVATS
AN4101.1  	M0--------------------------MVLFAPLAIFFINVISGSNEGVTIDYSLAAKS
          	*                           *.*:**::::** :***..     .*  .*.*

MG03399.1 	VAVFLGIPFGAAVITRFAIRATLGPEWFEKVFLRFISPWSLIGLLYTIVVLFASQGRQVV
NCU01852.1	VGVFLGMPLGAAILSRFTIRWLAGPVWFEKVFLRFAGPWSLIGLLYTILVLFASQGHAVV
FG06579.1 	VAVFLGIPLAAAIITRFSLRKLAGPEWYERVFLRIVSPWSLIGLLYTILILFASQGHQVV
AN4101.1  	VGVFLGIPLGAAILTRFALRLLISEEWYDRQFLKWLSPWSLIGLLFTILVLFASQGKQVV
          	*.****:*:.**:::**::*   .  *::: **:  .********:**::******: **

MG03399.1 	HQIVSVVRVAAPLTVYFFVIFFATLLVARRLRFGYALATTQSLTAASNNFELAIAVAVAT
NCU01852.1	HQIVSVLRVAAPLFVYFILIFSVTLYVTYRMGMGYALAATQSFTAASNNFELAIAVAVAT
FG06579.1 	HQIVSVVRVAAPLIVYFVVVFFATLLIARRAGFGYKLSTTQSFTAASNNFELAIAVAITA
AN4101.1  	HSIVSVVRVAAPLIVYFAVIFLVTLAVTRRFGFGYKLSCTQSFTAASNNFELAIAVAIAA
          	*.****:****** *** ::* .** :: *  :** *: ***:**************:::

MG03399.1 	YGPESDEALAATVGPLIEVPVLLALVYAVKYLGRRWTWK-
NCU01852.1	FGANSDQALAATVGPLIEVPVLIGLVYAVRWAGNRWAWKN
FG06579.1 	FGPSSDQALAATVGPLIEVPVLLGLVYLLRWVSDRWNWKI
AN4101.1  	FGVDSDQALAATVGPLIEVPVLLGLVYVVKWVARRQKWA-
          	:* .**:***************:.*** ::: . *  *
```
